# Estimating Colorectal Cancer Treatment Costs: A Pragmatic Approach Exemplified by Health Insurance Data from Germany

**DOI:** 10.1371/journal.pone.0088407

**Published:** 2014-02-19

**Authors:** Ulrike Haug, Susanne Engel, Frank Verheyen, Roland Linder

**Affiliations:** 1 Epidemiological Cancer Registry Baden-Wuerttemberg, German Cancer Research Center (DKFZ), Heidelberg, Germany; 2 WINEG – Scientific Institute of the Techniker Krankenkasse (TK) for Benefit and Efficiency in Health Care, Hamburg, Germany; Centro di Riferimento Oncologico, IRCCS National Cancer Institute, Italy

## Abstract

**Background:**

The cost of colorectal cancer (CRC) treatment is a crucial parameter to inform cost-effectiveness analyses on CRC screening but it is not readily available and therefore often lacking. We aimed to elaborate and exemplify a pragmatic approach to estimate CRC treatment cost based on health insurance data from Germany.

**Methods:**

We included two groups of persons who were continuously health-insured between 2005–2010: A) Cases: Persons with a hospital discharge diagnosis of CRC (ICD C18–C20) between 2007–2010 and no such a diagnosis between 2005–2006 (to focus on incident CRC cases); B) Controls: Persons without a diagnosis of CRC during the observation period, matched to CRC cases by age and sex (matching factor: 1∶5). We considered in-patient, out-patient and drug costs and calculated incremental costs as the difference in means between cases and controls. We divided costs into three phases of care (initial, intermediate and end-of-life phase).

**Results:**

The initial, the intermediate and the end-of-life phase included 12,792, 5,280, and 3,779 CRC cases, respectively, and 63,960, 26,400, and 18,895 controls. The mean incremental costs – annualized for each phase – were €26,000, €2,300, and €51,700, respectively. The costs of the initial phase of care were higher for rectal than for colon cancer. Annualized stage-specific cost estimates ranged from €15,000 to €21,300 for early stages and from €29,800 to €35,000 for late stages.

**Conclusion:**

This pragmatic and feasible approach provided plausible estimates of CRC treatment costs in Germany; being transferable to other settings, it may thus facilitate to weigh up potential savings in treatment costs against the resources required for CRC control programs in various countries.

## Introduction

Colorectal cancer (CRC) is the third most common cancer and cancer cause of death worldwide, with more than 1.2 million new cases and more than 600,000 deaths per year [1]. Randomized controlled trials have demonstrated the effectiveness of reducing CRC incidence and mortality by screening [2], [3]. Accordingly, several nations have already implemented a population-based CRC screening program, while others are still in the decision process or piloting phase [4], [5].

For the process of planning or piloting, but also for the evaluation and optimization of existing programs, cost-effectiveness analyses represent an important tool for health decision makers. On the cost side, such analyses weigh up expenditures for screening and costs due to complications against savings in treatment costs. Thus, cost on CRC treatment is a crucial parameter in such analyses, which, however, is not readily available.

In the ideal setting, this parameter would be estimated by following up a large and representative sample of newly diagnosed CRC patients and controls until death, with a direct linkage between detailed clinical information and health-related costs. However, this approach is complex, long-lasting and often not feasible due to resource constraints, technical hurdles or legal constrictions including data privacy. These challenges may explain why there is a lack of estimates on CRC treatment costs [6] and the need for detailed country-specific CRC cost studies has been highlighted [7]. Available estimates often originate from methodological approaches that are inconsistent or not transparent [8].

Accordingly, we aimed to elaborate a pragmatic approach to estimate CRC treatment cost from the health care payer perspective using health insurance data and exemplified this approach by data from Germany.

## Methods

We used data from the Techniker Krankenkasse (TK), the second-largest statutory health insurance in Germany that has members throughout Germany. The analysis is based on routinely collected data in agreement with the German social insurance act. Patient information was anonymized and de-identified prior to analysis.

Following previously described methods [9], [10] we included both CRC cases and controls to estimate net costs of CRC treatment and considered different phases of care, namely the initial phase of care (first 12 months after diagnosis), the intermediate phase of care and the end-of-life phase (last 12 months before death).

When selecting cases and controls for the analyses, we only considered persons who were continuously TK-insured from 2005 to 2010 or death, whichever came first, to avoid loss to follow-up due to change of the health insurance. Furthermore, we only considered principal insured persons and not co-insured family members given that for the latter the health insurance data are not complete with respect to date of death. From the remaining persons, we included the following to estimate costs for the initial and the intermediate phase of care:

- all persons with a hospital discharge diagnosis of CRC (ICD-10 C18–C20) between 2007–2010 and no such a diagnosis between 2005–2006 (to focus on incident CRC cases), and.
- a control sample consisting of randomly selected persons without a hospital discharge diagnosis of CRC during the period 2005–2010, matched to CRC cases by age and sex (matching factor: 1∶5).

To estimate costs for the end-of-life phase we only included CRC cases described under A) who died during the observation period and we randomly selected a control sample consisting of persons who were not diagnosed with CRC but died during the observation period, matched to CRC cases who died by age and sex (matching factor: 1∶5). To use the most up-to-date information for identifying subjects who are in the end-of-life phase, we extended the observation period to end of 2011 with respect to information on the vital status.

The matching and the subsequent analyses were done separately for ICD-10 C18 (colon), ICD-10 C19 (rectosigmoid junction) and ICD-10 C20 (rectum).

We tallied all in-patient, out-patient and drug costs occurring during the follow-up period for both CRC cases and controls. For CRC cases, follow-up started on the date of diagnosis and ended on the date of death or the end of the observation period (31 December 2010), whichever came first. For controls, we considered follow-up periods analogously to matched CRC cases. We calculated costs separately for the initial phase of care (first 12 months after the date of CRC diagnosis or after a corresponding surrogate date for controls), the end-of-life phase (last 12 months before the date of death) and the intermediate phase of care (all months in between). To ensure comparability we annualized the costs for the different phases.

To reduce the effect of outliers on the statistical analysis, we winsorized the cost estimates within each category before calculating means for the different diagnostic subgroups and phases of care. This is a commonly used transformation which reassigns the top and bottom percentile of a distribution (in this analysis: 5%) to the next lowest and highest values, respectively, counting inwards from the extremes [11].

Finally, we calculated incremental costs as the difference in means between CRC cases and controls for the different diagnostic subgroups (ICD-10 C18–20) and phases of care. We also calculated the incremental costs by phase of care for all three diagnostic subgroups combined.

We estimated ranges of costs for the initial phase of care stratified by early (UICC I and II) versus late stages (UICC III and IV), making assumptions on the distribution of CRC cases in Germany [12] and on the costs of the initial phase of care for early relative to late stages based on recent reports from other countries [13]–[16]. The formula to derive these estimates is explained in the **Appendix S1**.

The cost estimates of our analyses correspond to the value of Euros of the year 2009.

## Results

The number of CRC cases and controls included in the estimation of CRC treatment costs are shown in **Table 1** classified into phase of care and stratified by anatomical subsite (ICD-10 C18–20). Overall, the initial phase of care, the intermediate phase of care and the end-of-life phase included 12,792, 5,280, and 3,779 CRC cases, respectively, and five times the number of controls. Colon cancer cases accounted for the majority of patients in the three phases of care (63–68%). As expected according to the differences in CRC risk by gender, the majority of CRC patients were men. The proportions of men in the colon, the rectosigmoid, and the rectum cancer sample were 68%, 69%, and 75%, respectively, and the mean ages were 70.2, 69.8, and 69.5 years.

**Table 1:** Number of and mean costs among included CRC cases and controls overall and stratified by anatomical subsite classified into phase of care (the cost estimates are annualized as described in the methods section).

	Initial phase	Intermediate phase	End-of-life phase
**Colon cancer cases (ICD-10 C18)**			
N	8,638	3,550	2,387
Mean costs	27,735	5,145	68,947
**Controls matched to colon cancer cases**			
N	43,190	17,750	11,935
Mean costs	3,385	3,828	12,820
**Rectosigmoid cancer cases (ICD-10 C19)**			
N	657	276	271
Mean costs	32,705	8,957	58,631
**Controls matched to rectosigmoid cancer cases**			
N	3,285	1,380	1,355
Mean costs	4,055	4,229	13,374
**Rectal cancer cases (ICD-10 C20)**			
N	3,497	1,454	1,121
Mean costs	33,193	7,895	57,207
**Controls matched to rectal cancer cases**			
N	17,485	7,270	5,605
Mean costs	3,515	3,726	12,947
**Colorectal cancer cases (ICD-10 C18–20)**			
N	12,792	5,280	3,779
Mean costs	29.393	6.116	64.564
**Controls matched to colorectal cancer cases**			
N	63,960	26,400	18,895
Mean costs	3.451	3.821	12.886

Regarding the cost estimates (all cost estimates are annualized as described in the methods section), the mean costs in CRC patients showed peaks in the initial phase of care (€29,400) and the end-of-life phase (€64,600), while the mean costs in the intermediate phase of care were €6,100. In controls, the mean costs were also highest in the end-of-life phase (€12,900). In the time periods corresponding to the other phases the mean costs in controls were €3,500–3,800 (**Table 1**).

**Figure 1:**
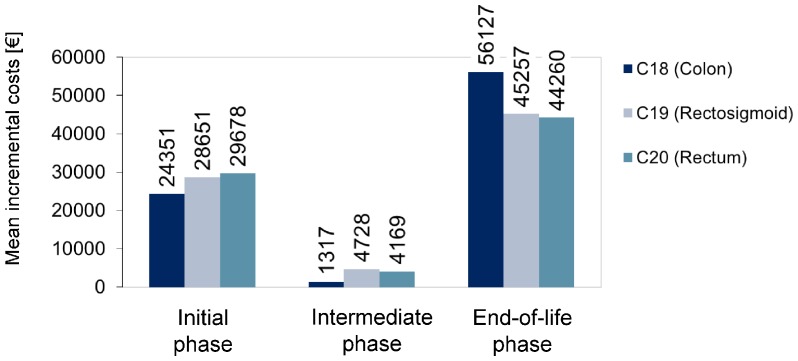
Mean incremental costs of CRC treatment classified into phase of care and stratified by anatomical subsite (the cost estimates are annualized as described in the methods section).

Overall, the mean incremental costs for the initial phase, the intermediate phase and the end-of-life phase were €25,900, €2,300, and €51,700, respectively. **Figure 1** displays the mean incremental costs classified into phase of care and stratified by anatomical subsite. The costs of the initial phase of care were, respectively, €4,300 and €5,300 higher for rectosigmoid and rectum cancer compared to colon cancer. Also, the costs of the intermediate phase of care were, respectively, €3,400 and €2,900 higher for rectosigmoid and rectum cancer compared to colon cancer. For the end-of-life phase, the costs were highest for colon cancer, with the difference amounting to €10,900 and €11,900 compared to rectosigmoid and rectum cancer, respectively.

**Table 2** shows estimates on CRC treatment costs regarding the initial phase of care stratified by early stages (UICC I and II) and late stages (UICC III and IV) using information on the cost ratio according to stage from the literature. The cost estimates ranged from €15,000 to €21,300 for early stages and from €29,800 to €35,000 for late stages.

**Table 2:** Estimates on CRC treatment costs (annualized) regarding the initial phase of care stratified by early stages (UICC I and II) and late stages (UICC III and IV) using information on cost ratios of late stages relative to early stages as reported from the literature (the formula to derive these estimates is explained in the Appendix S1).

Author, year; country(reference number)	Reported cost ratio oflate stages relativeto early stages	Estimated mean incremental costsfor early stages [€] assumingthe respective cost ratio	Estimated mean incremental costs for late stages [€] assuming the respective cost ratio
Tilson et al., 2013; Ireland [13]	1.40	21,264	29,770
Wilschut et al., 2011; Netherlands [14]	1.56	19,833	30,940
Lansdorp-Vogelaar et al., 2009; USA [15] 1	1.66	19,033	31,595
Clerc et al., 2008; France [16]	1.73	18,510	32,023
Landsdorp-Vogelaar et al., 2009;USA [15] 1	2.33	14,982	34,909

1 This study provided estimates on costs by stage for a present scenario, which are based on a data linkage study (ratio 1.66), as well as for a near-future scenario, which are based on expert opinion (ratio 2.33).

## Discussion

The cost of CRC treatment is a crucial parameter to inform cost-effectiveness analyses on strategies for primary prevention and early detection of CRC. Despite the timeliness and relevance of this topic, little attention has been paid to methodological approaches how to estimate this parameter [7]. In particular, methods are needed that satisfy the requirements of health-economic analyses without neglecting aspects of feasibility. We therefore elaborated a pragmatic approach to estimate mean incremental costs of CRC treatment by phase of care using health insurance data. Exemplified by data from Germany, our approach yielded (annualized) mean incremental costs of €26,000, €2,300, and €51,700 for the initial phase of care, the intermediate phase and the end-of-life phase, respectively, which are plausible in view of cost estimates reported from other countries [6]. The methodological approach can readily be used for repeated analyses to assess time trends in CRC treatment costs or be transferred to other settings.

Long-term follow-up of a cohort of incident CRC cases and matched controls whose clinical data are directly linked to health insurance data could be considered as the gold-standard of estimating CRC treatment costs. However, this approach is rather complex and clumsy [17]. Accordingly, cost estimates based on this approach are rarely available and if available, they are usually not up-to-date [6]. Simpler alternatives to this so-called incidence-based approach include the prevalence-based approach which measures the costs related to CRC during a given time period, with the underlying patient group being inherently heterogeneous. Although this approach provides information on total expenditure for the disease, it is of limited value for informing cost-effectiveness analyses on CRC prevention or early detection. The latter requires information, for example, on potential savings if CRC is prevented completely or if CRC death is prevented. Thus, a differentiation by phase of care is needed to satisfy the requirements of such analyses. Furthermore, cost estimates relative to a control group (incremental costs) rather than absolute costs are needed to take into account that the prevention of CRC or CRC death would not eliminate all health care spending in the respective individuals. Another simpler alternative is the estimation of CRC treatment costs solely based on treatment regimens, which is partly combined with modeling techniques to incorporate a time horizon and further information such as stage distribution. An important drawback of this approach is that there is no direct link to real-life costs, leaving the uncertainty whether the theoretical estimates meet the actual expenditures. Estimates on CRC treatment costs from the Netherlands that were solely based on rates of “Diagnose and Treatment Combinations” were strikingly lower compared to estimates based on real-life data from other countries [14].

In view of these alternatives, our approach appears to be a reasonable compromise between feasibility on the one hand, and meaningfulness and usefulness for informing cost-effectiveness analysis on the other hand. The comparison of our results with available estimates from Germany is hampered due to methodological differences. Two studies from 2007 estimated the average costs per CRC case. One study used a prevalence-based approach, yielding €21,820 per CRC case [18], the other approach was based on expert opinion, yielding €49,643 per CRC case [19]. To assess plausibility of our results, the comparison with estimates from other countries that also differentiated by phase of care may be helpful, although differences in the health care system and time periods need to be considered. A recent systematic review identified 10 cost-of-illness studies related to CRC, originating from France, the US, Ireland, and Taiwan [6]. In line with our results, the studies consistently showed that the initial phase of care and the terminal phase of care are the most expensive. For example, the net cost estimates used in US cost-effectiveness analysis corresponded to about $38,200, $3,100, and $49,000 for the initial, the intermediate and the end-of-life-phase across all stages (reported stage-specific estimates were averaged across all stages to facilitate comparison) [20]. Also, the finding that the initial phase of rectal cancer is more expensive than the initial phase of colon cancer is consistent with findings reported from other countries [13], [16], [21], which has been suggested to be attributable to (chemo)radiotherapy for rectal cancer patients.

While our estimates can directly be used for estimating the savings when CRC is prevented - for instance, by removal of precursor lesions or by primary prevention - the potential savings due to down-staging require further considerations. There are two main factors that may lead to savings when the disease is detected at an early as opposed to a late stage. First, early stages are less often recurrent or fatal, which saves expenditure following the initial treatment, in particular on terminal CRC care. To consider this aspect, it is important to classify cost estimates into phases of care including the end-of-life phase, as we have done in our analyses. Second, due to differences in treatment regimens the initial phase is less expensive for early stages as opposed to late stages [6]. To consider this aspect in cost-effectiveness analyses, stage-specific cost estimates are needed, which, however, were not available from the health insurance data as they lack information on stage of the disease. To overcome this limitation, we estimated a plausible range of stage-specific costs starting from overall CRC treatment and making assumptions on the distribution of CRC cases and costs by stage. The intention of this approach was to handle the uncertainty regarding stage-specific costs pragmatically by informing sensitivity analyses of later cost-effectiveness analyses that allow assessing whether or to which extent this uncertainty could influence the conclusion (that is, to assess robustness of the conclusion in this regard). We only differentiated by early stages (UICC I and II combined) versus late stages (UICC III and IV combined) given that the main aim of CRC screening programs is to avoid both advanced stages, that is stage III and stage IV. If needed for more detailed cost-effectiveness analyses, the approach could be extended to further differentiate by each single stage, requiring further assumptions in this regard. The prescription of targeted biological therapies is considered a main determinant of rising treatment costs for advanced CRC stages. Sales data of biological therapies from 2007 do not indicate that the use of these therapies in Germany is higher than in other countries [7], which justifies the approach of using cost ratios from other countries as point of orientation to estimate stage-specific costs for the initial phase of care.

With respect to the costs of the end-of-life phase estimated for CRC patients in our analysis, it needs to be taken into account that they refer to CRC patients who died within the temporary limited observation period. It can be expected that these patients have been diagnosed at an advanced stage for the most part. Theoretically, it is not clear why the end-of-life costs for CRC patients dying from the disease should differ according to the initial stage at diagnosis. However, they need to be distinguished from end-of-life costs for CRC patients dying from other causes. For the latter group, it seems reasonable to use the end-of-life costs estimated for control subjects when conducting cost-effectiveness analyses.

When interpreting our results, several aspects require consideration. First, it needs to be taken into account that the cost estimates have been derived in such a way to inform model-based cost-effectiveness analyses that simulate the development of CRC in a population under conditions with and without programs for CRC prevention and early detection [15], [20]. These cost estimates by phase of care cannot directly be translated into life-time costs of CRC, e.g. by summing them up. It matters how many months or years CRC patients stay in the different phases of care, which is considered by survival distributions in pertinent simulation models. Two examples may illustrate this: 1) not every CRC patient who dies of the disease will stay in the end-of-life phase for 12 months and cause the total costs of this phase. 2) CRC patients at stage III may have higher life-time costs than CRC patients at stage IV because they stay longer in the intermediate phase of care.

Second, to focus on incident CRC patients we excluded patients who had a hospital discharge diagnosis of CRC in 2005–2006, that is, two years before the actual observation period. With this criterion, patients who had a control examination in 2005–2006 due to CRC diagnosed before 2005 were also excluded. Although an extension of this “pre-observation period” would be desirable to minimize misclassification of prevalent cancer cases as incident cancer cases, this was not possible in our analyses because the health insurance data could be made available for five years only. It has been suggested that such a misclassification may result in an underestimation of the costs of the initial phase of care [22]. Two aspects may reduce the concern of having misclassified prevalent as incident CRC cases in our analyses. On the one hand, having had no control examination or any other disease-related treatment at all during the two years of pre-observation period appears rather unlikely for prevalent CRC cases. Furthermore, for CRC cases diagnosed in 2008 or later, the actual pre-observation period was longer than two years.

Third, although health care offers and reimbursements are for the most part uniformly regulated and priced across the various statutory health insurances in Germany, there might be some differences in the actual expenditure between health insurances, for example due to differential patterns of utilizing health care among insured persons. Validation of our results in terms of representativeness with data from another health insurance would certainly be of interest. Still, we expect that such potential differences in costs are minor and would not impact on the conclusion of cost-effectiveness analyses, in which the cost parameters are varied anyway within a certain range to assess robustness.

Fourth, we aimed to derive cost estimates on CRC treatment to inform cost-effectiveness analyses that are conducted from the payers’ perspective. Accordingly, the cost estimates do not include productivity loss which would be needed to address the societal perspective.

In conclusion, this pragmatic and feasible approach provided plausible estimates of CRC treatment costs that can be used for weighing up potential savings in treatment costs against the resources required for CRC prevention and screening. The methodological approach can readily be used for repeated analyses to assess time trends in CRC treatment costs and be transferred to other settings, which may finally enable various countries of the world to assess cost-effectiveness of CRC control programs.

## Supporting Information

Appendix S1 Formula used to derive the cost estimates for the initial phase of care by stage (early versus late stages).
(DOCX)
