# Supplementary material for: Estimating Colorectal Cancer Treatment Costs: A Pragmatic Approach Exemplified by Health Insurance Data from Germany
Source: PLoS One. 2014 Feb 19;9(2):e88407. doi: 10.1371/journal.pone.0088407 (PMC3929363; doi:10.1371/journal.pone.0088407)
Supplement: Appendix S1 — Formula used to derive the cost estimates for the initial phase of care by stage (early versus late stages). (DOCX) [file pone.0088407.s001.docx]

**Appendix S1:** Formula used to derive the cost estimates for the initial phase of care by stage (early versus late stages)

The cost estimates by stage were derived as follows:

Cost_AllS = CostES x PropES + CostES x PropLS x CostRatio,

corresponding to

Cost_AllS = CostES (PropES + PropLS x CostRatio),

which can be transformed to

CostES=Cost_AllS / (PropES + PropLS x CostRatio)

where

CostES denotes the estimated mean incremental costs for early stages,

Cost_AllS denotes the mean incremental cost across all stages, which was €25,942 for the initial phase of care in our analyses,

PropES denotes the proportion of CRC cases in Germany diagnosed at an early stage (UICC I and II), which was assumed to be 0.55 according to reference 12,

PropLS denotes the proportion of CRC cases in Germany diagnosed at a late stage (UICC III and IV), which was assumed to be 0.45 according to reference 12, and

CostRatio denotes the respective cost ratio of late stages relative to early stages as reported from the literature (see Table 2).

Once CostES is calculated, the cost of late stages (CostLS) can be calculated as follows:

CostLS = CostES x CostRatio
